# A Composite Structure of Modified Silver Nanoparticles for Improving the Recognition Performance of Electrode

**DOI:** 10.3390/mi17030384

**Published:** 2026-03-21

**Authors:** Jiao Yang, Liqin Cui, Yibo Zhao, Xiaoping Wu

**Affiliations:** 1 College of Microelectronics and Artificial Intelligence, Kaili University, Kaili 556011, China; 2 College of Science, Kaili University, Kaili 556011, China; 3 School of Optoelectronic Engineering, Xi’an Technological University, Xi’an 710021, China

**Keywords:** composite film, silver nanoparticle-modified, methylene blue, current response, aquatic products

## Abstract

To meet the demand for rapid detection of methylene blue residues in aquatic products, this study constructed a composite structure modified with silver nanoparticles on the surface of a glassy carbon sheet for precise detection. This composite film used the synergistic effect of the composite structure, which significantly enhanced the current response between the composite film and MB. The CV and EIS results demonstrated that this composite structure exhibited outstanding performance, endowing the composite film with the capability for sensitive detection of methyl blue. The results showed that the composite film detected methylene blue by differential pulse voltammetry, with a limit of detection as low as 1.6 nM. In the concentration range of 10 nM to 120 nM, the current intensity presented a good linear relationship with the concentration of MB. In addition, this composite film successfully identified methylene blue in aquatic products, with a recovery rate ranging from 81% to 113%. The results indicated that the composite film could be effectively applied to the sensitive detection of methylene blue in complex samples. This study provided a reliable and easy-to-construct electrochemical sensing platform for aquatic product safety monitoring.

## 1. Introduction

Methylene blue (MB) is a synthetic thiazine cationic dye. Due to its high bactericidal and antiparasitic properties, it was widely used in aquaculture to prevent and treat fish fungal and parasitic infections. However, MB concentrations exceeding the safe range may cause significant death of aquatic animals, and pose long-term pollution to the aquatic ecosystem [1,2]. Long-term human consumption of food containing MB residues can lead to increased neurotoxicity and carcinogenic risks [3]. Therefore, many countries and regions have explicitly banned its use in aquaculture. Unfortunately, due to its low cost and significant effect, illegal abuse still occurs from time to time. Therefore, developing a sensitive and reliable method for detecting methylmercury residues in aquatic products holds urgent practical significance for ensuring food safety and safeguarding human health.

At present, a large number of scholars designed different methods to detect MB, such as ultraviolet and visible spectroscopy [4], liquid chromatography coupled with tandem mass spectrometry [5], surface-enhanced Raman spectroscopy (SERS) and electrochemical methods [6,7]. Among them, SERS methods and electrochemical methods, due to their advantage of real-time detection, were widely used in the detection of MB. Vu et al. prepared silver nano-decahedra by photochemical method, and detected MB residues by SERS method with a limit of detection (LOD) of 10^−7^ M [8]. Wan et al. prepared an Au@TiO2 SERS sensor by ion sputtering and atomic layer deposition techniques, and its LOD for MB detection was 10^−6^ M [9]. Singh et al. prepared an anisotropic Au-Cu alloy nanostructured SERS sensor for MB detection, with an LOD of 10^−6^ M [10]. However, the SERS method still faced some challenges in practical applications, and its detection performance highly depended on the uniformity and reproducibility of noble metal (such as gold and silver) nanostructured substrates. Raman signals were easily interfered by the environment and sample matrix, and required high technical requirements for detection. In addition, SERS spectrometers were expensive, which was not conducive to technology popularization and on-site testing [11]. In contrast, electrochemical sensing technology, with its advantages of simple equipment, rapid analysis, low cost, easy miniaturization, and on-site integration, became an alternative for rapid MB detection.

At present, some scholars have successfully detected MB residues sensitively by electrochemical methods. Bahrololoomiet al. electrodeposited Ni-Fe on glassy carbon to form a composite electrode for MB detection, and the sensor had a LOD of 10^−5^ M for MB [12]. Bao et al. developed a self-supporting nitrogen-doped graphene electrochemical sensor, with a LOD of 9 × 10^−7^ M [13]. In addition, He et al. also reported an electrochemical sensor based on vertically ordered mesoporous silica channels, with a LOD of 4.1 × 10^−9^ nM for MB [1]. Soto et al. reported a self-doped TiO_2_ nanotube (SD-TNT) electrode for MB detection, and the SD-TNT electrode achieved efficient quantification of MB with a detection limit of 4.8 × 10^−6^ M [14]. However, the above-reported sensors still had room for improvement in the rapid MB detection performance. Therefore, it was necessary to design a high-performance electrode modification material that simultaneously achieved effective enrichment of target molecules, rapid electron transfer, and sensitive electrochemical response, thereby overcoming the limitations of existing detection technologies.

**Figure 1:**
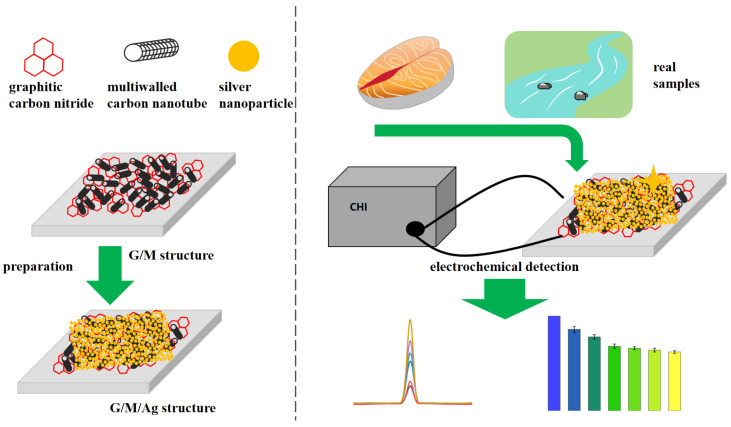
Electrochemical sensors with G/M/Ag structures prepared on GCE for detecting MB in aquatic products.

As shown in Figure 1, this study designed and prepared a graphitic carbon nitride/multi-walled carbon nanotube/silver nanoparticle composite structure on a glassy carbon electrode to construct an electrochemical sensor (GCE/G/M/Ag). This composite structure fully integrated the synergistic advantages of different materials. The nitrogen-rich layered structure of g-C_3_N_4_ could provide abundant binding sites, enabling the adsorption and pre-enrichment of methylene blue [15]. The three-dimensional conductive network constructed by MWCNTs could promote the rapid transmission of electrons inside the composite structure, thereby effectively improving the conductivity of the sensor [16]. Moreover, the highly dispersed Ag nanoparticles on the sensor surface could further enhance the overall conductivity, thereby amplifying the electrochemical response signal after the sensor reacted with molecules. The experimental results indicated that the GCE/G/M/Ag sensor exhibited a low LOD for MB, as low as 1.6 nM. This sensor was successfully applied to the electrochemical detection of MB residues in actual aquatic product (fish tissue, river water) samples. The excellent detection performance of the GCE/G/M/Ag sensor provided a reliable reference scheme for the development of portable detection equipment suitable for on-site rapid screening.

## 2. Materials and Methods

### 2.1. Products

PBS, ethanol, aluminum oxide, graphitic carbon nitride, multiwalled carbon nanotubes, AgNO_3_, citric acid, Na_2_SO_4_, and NaF were obtained from Sinopharm Chemical Reagent Co., Ltd. (Shanghai, China). The glassy carbon sheet was purchased from Shanghai Sanshe Industrial Co., Ltd. (Shanghai, China).

### 2.2. Instrumentation

To characterize the surface structure of the sensor and investigate its electrochemical properties, scanning electron microscopy (SEM, SU1510, Hitachi, Ltd., Tokyo, Japan) was employed to observe the morphology of the surface of sensor. Comprehensive electrochemical testing was conducted using an electrochemical workstation (CHI 660C, Shanghai, China) to analyze the interaction and response behavior between the sensor and methyl blue molecules.

### 2.3. Synthesis of GCE/G/M/Ag Sensor

First, the bare GCE was pretreated, and its surface was carefully polished with 0.03 μm alumina powder. After the polishing operation was completed, the electrode surface was thoroughly rinsed with distilled water, and then it was placed in air to dry naturally. Next, the required electrolyte was prepared, which contained 1 g/L graphitic carbon nitride, 0.5 g/L multiwalled carbon nanotubes, 1 mol/L sodium sulfate (Na_2_SO_4_) and 0.5 wt% sodium fluoride. With GCE as the anode and Pt as the cathode, deposition was carried out under a constant voltage of 10 V. After 40 min of deposition, the GCE/G/M film was obtained [17]. Subsequently, the prepared GCE/G/M film was immersed in an electrolyte containing 1 mM silver nitrate (AgNO_3_) and 0.1 M citric acid. With the GCE/G/M film as the anode and Pt as the cathode, deposition was carried out at a potential of −0.7 V for 30 min, and finally the GCE/G/M/Ag sensor was prepared [18].

### 2.4. Parameters for Electrochemical Testing

In this work, Cyclic voltammetry (CV) curves of different structures were tested in PBS with pH 7. The potential range was −0.2 V to 1.2 V, and the scan rate was 50 mV/s. The test frequency of electrochemical impedance spectroscopy (EIS) was 10^−1^ to 10^5^ Hz, and the amplitude was 50 mV. In this study, the potential range for detecting MB by the differential pulse voltammetry (DPV) method was −0.8 V to 0.2 V, and the amplitude was 50 mV.

### 2.5. Treatment of Real Samples

We purchased grass carp tissues from the local HeLi Supermarket. First, the fish tissues were pretreated according to the literature [19]. After that, each sample was homogenized, and 1 mL of 1 μM MB solution was added to 5 g of the above samples. After centrifugation at 3000 rpm for 5 min, the supernatant was collected and added to PBS for testing. In addition, we collected water from the Bala River and added MB into the river water while stirring evenly. Then it was added to PBS to prepare river water samples with different concentrations.

## 3. Results

### 3.1. Morphological Characterization of SEM

**Figure 2:**
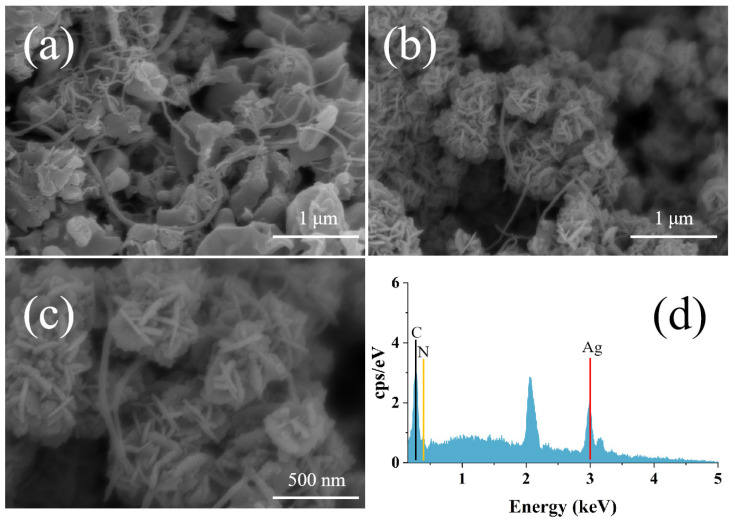
(**a**) The surface morphology of the G/M structure deposited on the GCE in the scanning electron microscope. (**b**) The surface morphology of the GCE/G/M/Ag structure in the scanning electron microscope. (**c**) A local magnified view of the morphology of the GCE/G/M/Ag structure. (**d**) EDS result of GCE/G/M/Ag structure.

As shown in Figure 2a, g-C_3_N_4_ nanosheets were intertwined with MWCNTs, filling the stacking gaps of g-C_3_N_4_. MWCNTs themselves had excellent conductivity and a one-dimensional nanostructure, which could construct continuous electron transport channels. This material could effectively reduce the transport resistance of electrons in the composite system, further improving the response rate of the sensor. At the same time, its high specific surface area also provided additional sites for the adsorption of MB molecules. In electrochemical tests, this composite structure could adsorb MB molecules in the solution through the electrostatic interaction of g-C_3_N_4_, increasing the local concentration of MB at the sensor interface. As shown in Figure 2b, silver nanoparticles (AgNPs) were attached to the defect sites of g-C_3_N_4_, as well as the tube walls and cross-linking nodes of MWCNTs, forming high-density nanoscale electrocatalytic active sites. The nanoscale stacking structure of AgNPs could be clearly seen in Figure 2c, which facilitated the capture of MB molecules in the solution. In addition, AgNPs had strong conductivity, which could sensitively reflect the molecular concentration on the sensor surface, forming a synergistic effect with g-C_3_N_4_ and MWCNTs to significantly optimize the detection performance of the sensor. The result in Figure 2d showed the EDS spectrum of the GCE/G/M/Ag sensor. It was clearly observed that the sensor primarily consisted of C, N, and Ag elements. The C element originated from the GCE, g-C_3_N_4_ and MWCNTs, the N element from g-C_3_N_4_, and the Ag element from silver nanoparticles. These results confirmed the formation of the GCE/G/M/Ag composite structure.

### 3.2. Electrochemical Performance Testing of GCE/G/M/Ag Sensor

**Figure 3:**
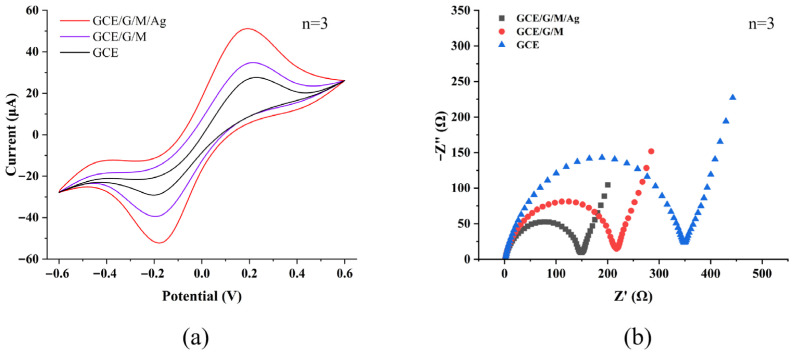

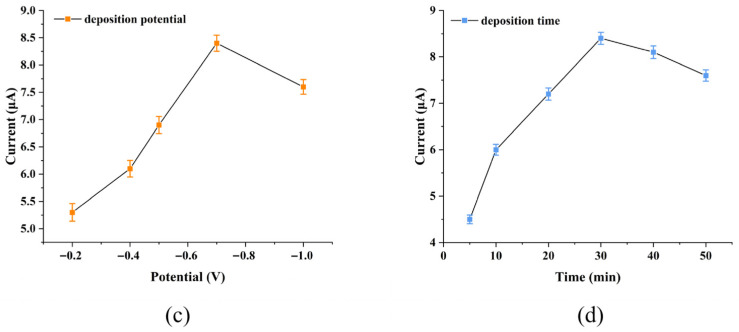
(**a**) CV curves of GCE, GCE/G/M and GCE/G/M/Ag structures in 1.0 mM K_3/4_Fe(CN)_6_ with 0.1 M KCl. (**b**) EIS curves of GCE, GCE/G/M and GCE/G/M/Ag structure in PBS. (**c**) The influence of different Ag deposition potentials on the detection of MB. (**d**) The influence of different Ag deposition times on the detection of MB.

The curves in Figure 3a were the results of the CV tests of three different structured electrodes (GCE, GCE/G/M and GCE/G/M/Ag) in K_3/4_Fe(CN)_6_ with 0.1 M KCl. The potential range was controlled at −0.6~1.6 V and the scan rate was set at 50 mV/s during the test. The curve data in Figure 3 represented the approximate average value obtained from three repeated CV tests using the electrochemical workstation. It could be found that only the GCE/G/M/Ag structured electrode showed obvious redox peaks, because the AgNPs loaded in the composite structure had strong oxidizability and electrochemical activity, which could significantly promote electron transfer. The Randles-Sevick equation was used to calculate the electroactive surface area of the GCE/G/M/Ag film:

I_P_ = 2.69 × 10^5^An^3/2^D^1/2^Cv^1/2^ (1)

Here, I_P_ represents the peak current of the sensor, D denotes the diffusion coefficient of [Fe(CN)_6_]^3−/4−^, v is the scan rate, A is the electroactive surface area of the electrode, and C is the concentration of the [Fe(CN)_6_]^3−/4−^ solution. The electroactive surface area of the GCE/G/M/Ag film was calculated to be 0.3 cm^2^.

To further study the electrochemical properties of the substrate, this experiment tested the charge transfer resistance (Rct) on the surface of different structures by EIS, and the test frequency range was set at 10^−1^~10^5^ Hz. The EIS curve results in Figure 3b showed that the pure GCE had the largest Rct value, indicating that it had the strongest interface electron transfer resistance. The GCE/G/M/Ag structured electrode had the smallest Rct value, which further indicated that the composite structure had stronger electrochemical activity. This was because the porous nanostructure formed by the GCE/G/M film had a large specific surface area, which could provide a large number of active sites for the uniform deposition of AgNPs. This structure effectively reduced the agglomeration of AgNPs, and thus significantly improved the electrochemical activity and interface charge transfer efficiency of the sensor. In addition, the performance of the sensor was highly dependent on the Ag deposition conditions. A deposition potential that was too positive did not reduce Ag^+^ onto the composite, while a potential that was too negative led to the rapid, uncontrolled formation of stacked Ag aggregates, which hindered electron transfer. Deposition time was equally critical. When the time was too short, the catalytic effect was weak due to low Ag loading. When it was too long, the Ag nanoparticles agglomerated, blocking active sites on the g-C_3_N_4_/MWCNT composite and reducing the effective surface area. By testing the electrochemical response to 50 nM MB, the optimal parameters were identified as a deposition potential of −0.7 V for 30 min, which produced the highest peak current.

**Figure 4:**
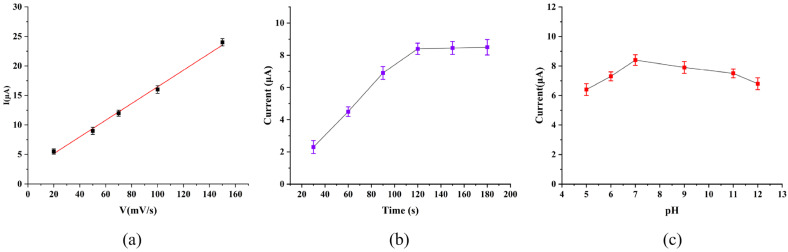
(**a**) CV peak current intensity of GCE/G/M/Ag structures at different scanning rates. (**b**) CV peak current intensity of GCE/G/M/Ag structures at different scanning rates. The influence of enrichment time on the detection of MB by GCE/G/M/Ag sensor. (**c**) The effect of solution pH on the detection of MB by GCE/G/M/Ag sensor.

In this work, we investigated the adsorption of GCE/G/M/Ag films on MB molecules at a concentration of 50 nM. As shown in Figure 4a, as the CV sweep rate increased, the oxidation peak intensity of MB molecules showed a good linear relationship with the sweep rate within the range of 20 to 150 mV/s. This indicated that the electrode process was mainly controlled by adsorption [6]. Before carrying out electrochemical tests, the sensor needed to be pretreated. First, the sensor was immersed in a standard solution containing 50 nM MB, and a potential of −0.1 V was applied to the sensor to promote the adsorption of MB molecules in the solution on the sensor surface. The results in Figure 4a showed that the sensor’s response to MB increased rapidly in the first 90 s, and the growth rate slowed down from 90 to 120 s. When the applied potential lasted for 120 s, the current response of the sensor to MB reached the peak, indicating that MB molecules had been fully adsorbed on the sensor surface and the adsorption reached saturation. This current saturation phenomenon was attributed to surface adsorption-induced confinement effects that restrict electron transport dynamics. In addition, the pH value of the solution had a significant impact on the electrochemical activity of the measured molecules. MB molecules were dissolved in PBS with different pH values respectively, and detected sequentially by the sensor. As shown in Figure 4b, when the solution pH was 7, the current response of the sensor to the MB molecules was the most significant. Therefore, in this work, the enrichment time of the GCE/G/M/Ag sensor for MB detection was 120 s, and the pH of the selected electrolyte was 7.

**Figure 5:**
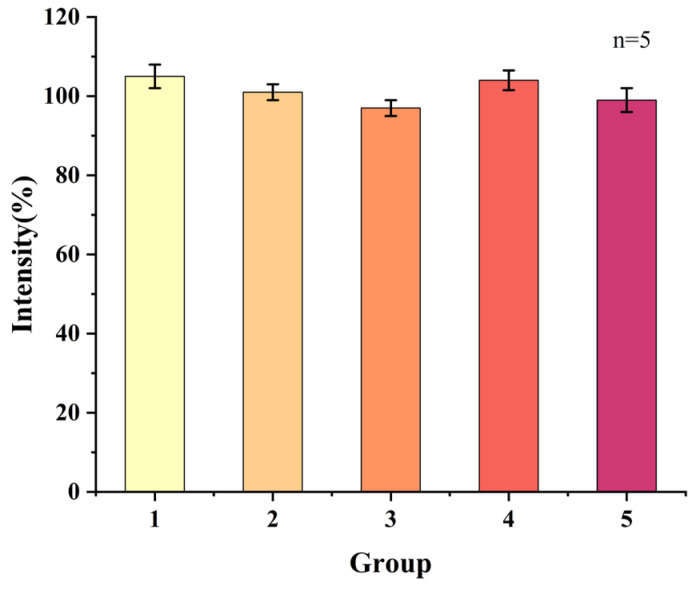
Reproducibility test of GCE/G/M/Ag sensor for detecting MB.

To systematically evaluate the detection repeatability of the sensor for MB molecules, this study used five groups of independent sensors (each group contained five parallel samples) to detect MB solutions of the same concentration. Figure 5 showed the current intensities collected by each group of sensors for MB detection. The results showed that the current signals obtained by the five groups of sensors had high consistency, and their calculated relative standard deviation (RSD) was 6.8%. This data indicated that the prepared GCE/G/M/Ag sensor had good repeatability and measurement stability in MB detection, which could meet the reproducibility requirements of actual detection.

### 3.3. GCE//G/M/Ag Sensor Detects MB Molecules Using the DPV Method

**Figure 6:**
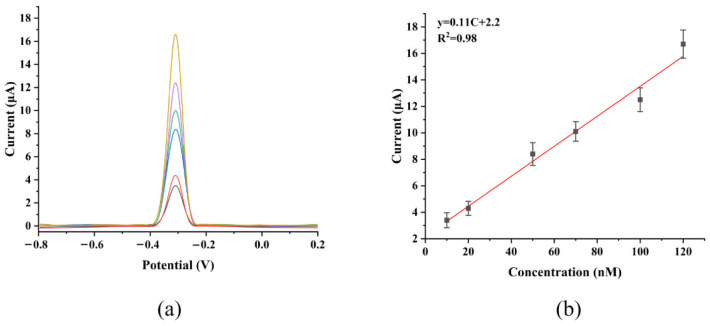
(**a**) The sensor uses DPV to detect MB molecules of different concentrations. (**b**) The electrochemical sensitivity of the GCE/G/M/Ag sensor to MB molecules.

As shown in Figure 6a, under room temperature test conditions, the electrochemical response current of the GCE/G/M/Ag sensor increased significantly with the increase in MB concentration when differential pulse voltammetry (DPV) was used for point-by-point testing. The signal change was clear and stable, demonstrating excellent detection reliability. This phenomenon was mainly attributed to the efficient adsorption of MB molecules by the abundant active sites on the sensor surface. This adsorption led to an increase in the local concentration of reactants at the electrode interface, thereby effectively improving the electron transfer efficiency and electrochemical response signal. In addition, the synergistic effect formed by the composite structure further accelerated the charge transfer rate on the sensor surface, thus further improving the electrochemical performance. According to the fitting results of the DPV data in Figure 6b, a good linear relationship was observed between the oxidation peak current intensity and MB concentration in the range of 10 nM to 120 nM. The correlation coefficient (R^2^) reached 0.98, indicating that the sensor possessed strong quantitative analysis capability for MB detection within this linear range. The calculation result showed that the LOD of the GCE/G/M/Ag sensor for MB could reach 1.6 nM, showing excellent detection sensitivity. These above results indicated that the GCE/G/M/Ag sensor had high sensitivity, good linear response and low detection limit for MB detection, and these advantages provided strong support for its application in real scenarios.

### 3.4. The Selective Recognition Ability of the GCE/G/M/Ag Sensor for MB Molecules

**Figure 7:**
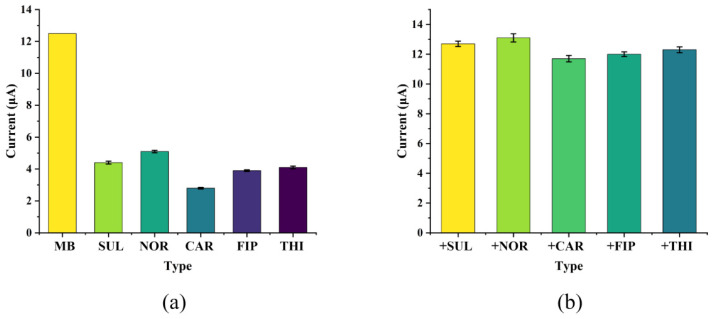
(**a**) The electrochemical response intensity of the GCE/G/M/Ag sensor to different molecules. (**b**) The electrochemical response intensity of the GCE/G/M/Ag sensor in the presence of interfering solutions for detecting MB molecules.

To further evaluate the anti-interference performance of the sensor, this study selected five different molecules (sulfamethoxazole, norfloxacin, carbaryl, fipronil, thiabendazole) as interfering substances, and carried out corresponding anti-interference experiments. Figure 7a showed the electrochemical response intensity of the GCE/G/M/Ag sensor to different molecules, and it could be found that the sensor had obvious response intensity to MB. After that, MB and each interfering substance were dissolved in phosphate buffer solution (PBS) respectively to prepare mixed solutions containing 10 nM MB and 50 nM interfering substances. Then, the GCE/G/M/Ag sensor was immersed in the mixed solution for electrochemical testing, and the analysis was carried out by recording the peak current of the DPV curve. The result was shown in Figure 7b. In the presence of interfering substances, the DPV peak current intensity detected by the sensor was very close to that obtained from the test with the MB standard solution. Compared with the standard solution containing only MB, its electrochemical response error was kept within 10%. This result indicated that the GCE/G/M/Ag sensor had good selective recognition ability for MB molecules. This was because g-C_3_N_4_ could efficiently adsorb and enrich target molecules through π-π stacking interactions [20]. Furthermore, methylene blue existed as a cation in aqueous solutions. At an enrichment potential of −0.1 V, the g-C_3_N_4_ composite structure preferentially captured MB molecules, effectively mitigating interference from other substances.

### 3.5. GCE/G/M/Ag Sensor Detect MB Molecules in Aquatic Products

**Figure 8:**
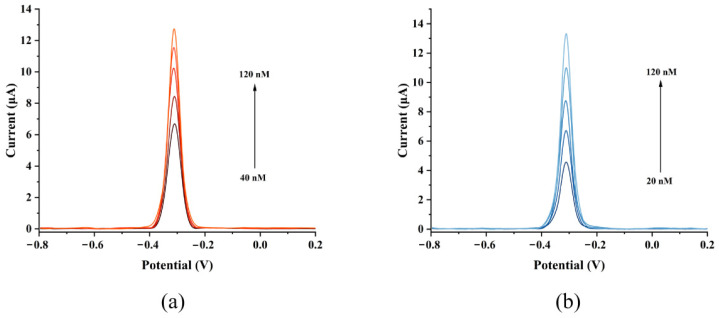

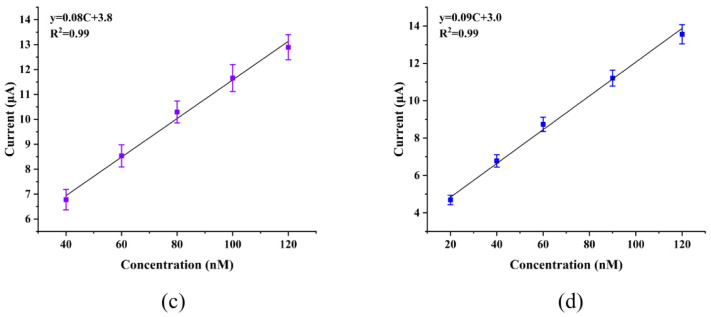
(**a**) GCE/G/M/Ag sensor detecting MB on fish tissue. (**b**) GCE/G/M/Ag sensor detecting MB in river water. (**c**) Sensitivity of the sensor detecting MB in fish tissue. (**d**) Sensitivity of the sensor detecting MB in river water.

To verify the practical application performance of the prepared GCE/G/M/Ag sensor, this study selected fish tissue and river water as real samples for electrochemical testing research. We added MB molecules to the two pretreated types of sample solutions to prepare a series of real spiked samples with different MB concentrations to simulate the real detection environment. As shown in Figure 8a,b, the sensor exhibited clear DPV current responses in complex real samples. The results in Figure 8c,d showed that the electrochemical response of the GCE/G/M/Ag sensor to MB concentration in both matrices had a good linear relationship. It was found that the sensor exhibited a higher LOD for MB in fish tissue samples (40 nM) compared to that in river water (20 nM). This discrepancy was primarily attributed to the necessary sample pretreatment process. The extraction and centrifugation steps, while effective at removing matrix interferences, inevitably introduced a dilution effect. This dilution reduced the final concentration of MB introduced into the electrochemical cell, thereby limiting the ability of the sensor to detect MB in fish tissue.

Table 1 listed the spiked recovery rates of the GCE/G/M/Ag sensor for MB detection in actual samples. The data showed that high recovery rates of MB were achieved in both fish skin tissue and river water samples within the detection concentration range, indicating accurate and reliable detection results. The above results proved that the GCE/G/M/Ag sensor had reliable analytical performance in complex actual samples and strong applicability in real environments.

**Figure 9:**
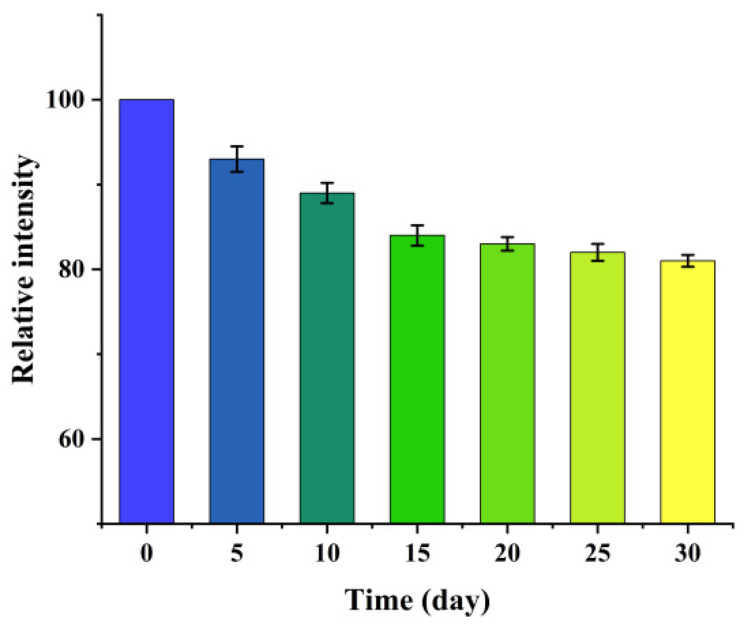
Stability testing of MB molecules using GCE/C/M/Ag sensor.

In addition, to evaluate the long-term storage stability of the sensor we stored it in a sealed environment at room temperature and away from light for 30 days. We tested its sensing performance multiple times at fixed time intervals. As shown in Figure 9, after 30 days of long-term storage, the detection intensity of the sensor for MB could still maintain about 84% of the initial detection intensity, and the performance attenuation was small. The above experimental results indicated that the prepared composite structure possessed excellent storage stability and service durability. It could meet the application requirements for long-term sensing detection environment.

To compare the performance of the sensor prepared in this paper, Table 2 listed the main parameters of different current sensors for MB molecule detection. It facilitated the intuitive comparison of the detection capabilities of different sensors. The results clearly showed that the LOD of the GCE/G/M/Ag sensor was much lower than that of other sensors, and it had significant advantages in the detection of low-concentration analytes. This was because the synergistic effect within the composite material endowed the sensor with strong and stable detection capability, effectively enhancing its signal response and molecular recognition ability. These properties enabled the sensor to achieve high sensitivity and selectivity in detecting at extremely low concentrations. The sensor demonstrated broad and promising application prospects in environmental monitoring, food safety, and other practical analytical fields.

## 4. Conclusions

This study fabricated an electrochemical sensor based on a GCE/C/M/Ag composite structure, and it was used for sensitive and rapid detection of methylene blue residues in aquatic products. The synergistic effect of nanocomposites greatly improved its detection performance, and it achieved efficient determination of methylene blue. CV and EIS results proved the excellent electrochemical activity of the composite structure, and the DPV current showed good linearity with MB concentration from 10 to 120 nM. The LOD was as low as 1.6 nM, and the sensor kept obvious electrochemical response to MB under high-concentration interferences. It exhibited high selectivity in anti-interference tests, and the sensor successfully detected MB residues in fish and river water samples. The recovery rates of real samples tests met the expected requirements. This work established a facile, rapid, and highly sensitive sensor for aquatic product monitoring, demonstrating considerable practical application potential. In order to make this sensor applicable to other harmful dyes, in future work we will modify the sensor through methods such as antibody/aptamer functionalization and molecularly imprinted polymers to enable the detection of different molecules.

## Figures and Tables

**Table 1:** The GCE/G/M/Ag sensors measured the MB recovery rate in fish tissue and river water, respectively.

Sample	Add (nM)	Detect (nM)	Recovery (%)	RSD (*n* = 5)
fish	40	42.3	105.7	6.1
	60	57.7	96.2	5.2
	80	74.2	92.8	4.2
	100	86.1	86.1	4.6
	120	97.9	81.6	3.9
river	20	22.7	113.5	5.4
	40	41.7	104.3	4.9
	60	59.4	99.0	4.4
	90	82.5	91.7	3.8
	120	103.9	86.6	3.8

**Table 2:** Key parameters for detecting MB using different sensors.

Methods	Sensor	LOD (mol/L)	References
SERS	Silver nano-decahedron	10^−7^	[8]
SERS	Au @ TiO2	10^−6^	[9]
SERS	Au-Cu alloy nanostructure	10^−6^	[10]
Electrochemistry	GCE/Ni-Fe	10^−5^	[12]
Electrochemistry	Self-supporting nitrogen-doped graphene	9 × 10^−7^	[13]
Electrochemistry	Silicon dioxide channel	4.1 × 10^−9^	[1]
Electrochemistry	SD-TNT	4.8 × 10^−6^	[14]
Electrochemistry	GCE/G/M/Ag	1.6 × 10	This study

## Data Availability

The original contributions presented in this study are included in the article. Further inquiries can be directed to the corresponding author.
